# Abstract of Proceedings of Buffalo Medical Association

**Published:** 1866

**Authors:** Thos. M. Johnson

**Affiliations:** Sec’y.


ART. II.—*Abstract of Proceedings of Buffalo Medical Association.*

TUESDAY EVENING, February 6, 1866.

Association met pursuant to adjournment and was called to order by the President, Dr. Ring. Present—Drs. Ring, Gay, Strong, Brown, Rochester, Boardman and Johnson.

DR. GAY exhibited a vesical calculus which he had removed by the usual operation. The stone was egg shaped, and weighed half an ounce. Prof. Hadley bisected it, and on chemical examination found its interior portion to consist of oxalate of lime, and its exterior rough portion to consist of the phosphates. It had been a source of great irritation for six years. The distance from the surface of the perineum to the neck of the bladder was much greater than usual. The index finger passing up through the wound could barely be made to reach the neck. The patient was sixty-eight years of age, and died on the sixth day after the operation.

DR. ROCHESTER reported the case of a young man to whom he was called, and found on inquiry that he had had all the symptoms of small pox. On examination found great dysnæ, also distinct and profuse purpural eruption. Next morning found the distinct small pox eruption. Directed him to take five drops sol. subsephate ferri every two hours. He is now at the ninth day of the eruption and doing well. This was not erythema nor was it roseola. Had seen one case of malignant small pox previous to the case just reported.

Dr. Strong reported a case of rubeola with hæmatemesis preceding the eruption. The hæmorrhage was arrested with per sulph. ferri, and the case resulted favorably.

Miscellaneous business being in order,

Dr. Rochester read a communication from the Council of Hygiene and Public Health, of New York city, asking the profession of this city to take such action as may seem most judicious for the prevention of epidemic cholera if it shall prevail here at any time in the future.

After considerable discussion upon this subject and the means to be adopted for carrying forward preventive measures, Dr. Rochester offered the following, which was unanimously adopted:

*Resolved*, That every member of the regular profession, resident in Buffalo and vicinity, is earnestly invited to be present at the rooms of the Buffalo Medical Association on Tuesday, February 13, 1866, at 7 o'clock P. M. to take action respecting the sanitary measures proper to be instituted for the welfare and protection of our citizens during the ensuing summer.

Dr. Boardman made the following report of the soldiers received into and treated either medically or surgically at the Buffalo Hospital of the Sisters of Charity, compiled by M. Picket, remarking that he believed that the results of treatment in that institution had been very favorable:

Whole number received from June 30th, 1864 to August 31st, 1865—14 months. Whole number of surgical cases 186; whole number of medical cases 177; total 363; 74 had chronic diarrhœa; deaths from surgical cases 4; chronic diarrhœa 3; small pox 1; other medical diseases 3; total 11; whole number returned to duty 125; discharged or mustered out of service 123; transferred to other hospitals 51; reported deserted, but of whom many returned and were afterwards honorably mustered out 53; not accounted for 11. About twenty of these were sent to the front, and were a second time sent to this hospital on account of some medical or surgical disease contracted since their first discharge from this institution.

Dr. Strong remarked that he believed that the cases mentioned above were, with but few exceptions, mild—not severely sick—and that the good results reported were to be expected.

Dr. Boardman said that many of the cases were very severe, and regards the results as very flattering.

Dr. Gay concurs in Dr. Boardman's statements.

Report on prevailing diseases being called for variola and varioloid were reported as prevailing.

Adjourned. Thos. M. Johnson, *Sec'y.*

